# Neurodevelopmental outcome of low-risk moderate to late preterm infants at 18 months

**DOI:** 10.3389/fped.2023.1256872

**Published:** 2023-11-30

**Authors:** Mary Anne Ryan, Deirdre M. Murray, Eugene M. Dempsey, Sean R. Mathieson, Vicki Livingstone, Geraldine B. Boylan

**Affiliations:** ^1^ INFANT Centre, University College Cork, Cork, Ireland; ^2^ Department of Paediatrics and Child Health, University College Cork, Cork, Ireland; ^3^ Department of Neonatology, Cork University Maternity Hospital, Cork, Ireland

**Keywords:** preterm birth, premature infant, moderate to late preterm, developmental outcomes, Griffiths III

## Abstract

**Background:**

Of the 15 million preterm births that occur worldwide each year, approximately 80% occur between 32 and 36 + 6 weeks gestational age (GA) and are defined as moderate to late preterm (MLP) infants. This percentage substantiates a need for a better understanding of the neurodevelopmental outcome of this group

**Aim:**

To describe neurodevelopmental outcome at 18 months in a cohort of healthy low-risk MLP infants admitted to the neonatal unit at birth and to compare the neurodevelopmental outcome to that of a healthy term-born infant group.

**Study design and method:**

This single-centre observational study compared the neurodevelopmental outcome of healthy MLP infants to a group of healthy term control (TC) infants recruited during the same period using the Griffith's III assessment at 18 months.

**Results:**

Seventy-five MLP infants and 92 TC infants were included. MLP infants scored significantly lower in the subscales: Eye-hand coordination (C), Personal, Social and Emotional Development (D), Gross Motor Development (E) and General Developmental (GD) (*p* < 0.001 for each) and Foundations of Learning (A), (*p* = 0.004) in comparison to the TC infant group with Cohen's *d* effect sizes ranging from 0.460 to 0.665. There was no statistically significant difference in mean scores achieved in subscale B: Language and Communication between groups (*p* = 0.107).

**Conclusion:**

MLP infants are at risk of suboptimal neurodevelopmental outcomes. Greater surveillance of the neurodevelopmental trajectory of this group of at-risk preterm infants is required.

## Introduction

The World Health Organisation (WHO) has acknowledged that whilst reducing mortality for newborns is the priority there is also a need to prioritise improving health, psychosocial well-being and the learning potential of children, particularly in the early years of life (1). Over the past decades improvement in NICU care and technology has led to increased survival rates of preterm infants. However, despite this success, gains in survival have not been paralleled by improvements in morbidity.

Of the 15 million preterm births that occur worldwide each year, approximately 75% to 84% occur between 32 and 36 + 6 weeks gestational age (GA) and these infants are defined as moderate to late preterm (MLP) (2, 3). Current trends have shown that the rise in the rate of preterm birth is primarily attributed to the MLP infant group (4). This may in part reflect the increased use of artificial reproductive technology and its association with multiple pregnancies and preterm delivery and consequently an increased demand for neonatal unit services (5).

In the last six weeks of pregnancy, there is a 35% to 40% increase in brain weight with a five-fold increase in brain volume (6). After birth, the MLP infant is no longer protected by the uterine environment and this critical period of accelerated development takes place externally in the NICU or postnatal wards (7). A challenging external environment may influence neural connectivity (8). Disruption of the typical process of brain development may expose preterm infants to long-term neurodevelopmental impairment. Whilst a more positive outcome is associated with the low-risk uncomplicated preterm infant (9, 10) defining “uncomplicated” may prove problematic as preterm birth itself is atypical and its aetiology may involve deteriorating fetal or maternal health, which in itself may lead to suboptimal outcomes. Maturity at birth has a direct impact on outcome; the lower the GA of the preterm infant, the greater the impact on mortality and morbidity (11–14). Low birth weight associated with preterm birth further increases risk factors for the preterm infant (15).

The availability of neurodevelopmental outcome data for healthy MLP infants is often hindered by the variation in follow-up between institutions internationally (16) and the belief that these infants are low risk, and hence require little developmental surveillance. This limits our ability to assess the prevalence of impairments in this healthy MLP infant group. This study aims to establish the natural neurodevelopmental baseline scores for a healthy homogenous group of MLP infants admitted to the neonatal unit at birth. Secondly, we aim to compare the neurodevelopmental outcome of the MLP group to that of a healthy term-born infant's group at 18 months postmenstrual age, recruited during the same period.

## Methods

Cork University Maternity Hospital is the largest Maternity Hospital in the south of the Republic of Ireland, serving the local and surrounding areas, and is a referral centre for high risk pregnancies.

### Participants

All participating MLP infants were recruited from the neonatal unit between July 2017 and September 2018 as part of a single-centre observational sleep study. Inclusion criteria required infants to be born between 32 and 36 + 6 weeks GA, admitted to the neonatal unit at birth, a normal physical/neurological exam for GA conducted by the clinical team at birth, clinically stable, no actual or suspected chromosomal abnormalities and a normal cranial ultrasound if performed.

As this study places a narrow focus on the natural neurodevelopment of a healthy MLP infant group, this study specifically excluded infants with a complicated neonatal stay requiring an escalation in medical care including surgery, treatment of sepsis, requiring prolonged invasive respiratory support, requiring a blood transfusion or persistent hypoglycaemia or hyponatraemia. There is no universally accepted threshold for safe blood glucose concentrations (17) despite the fact that hypoglycaemia has been identified as a risk factor for suboptimal development (18). No MLP infant had a diagnosis of hypoglycaemia based on discharge summary reports. Transient hypoglycaemia may have occurred as part of the normal adaption to extrauterine life (19) in some infants but hypoglycaemia was not a concern for any MLP infant (local protocol defines hypoglycaemia as laboratory blood sugar level <2.8 mmol/L (45 mg/dl).

Infants requiring treatment for physiological jaundice which resolved with phototherapy were included in this study and were identified in the discharge summary report. No MLP infant required prolonged phototherapy or phototherapy for reasons other than normal physiological jaundice. All infant's enteral feeds progressed in volume as per local feeding regime and were tolerated well, with full oral feeds (breast or bottle) established for a minimum of 48 h prior to discharge. In 2016, the year prior to the commencement of this study, 64,097 infants were born in Ireland and 3.8% were multiple births. Forty six percent of multiple births were born between 32 and 36 weeks GA (20). We therefore included MLP infants from singleton and multiple pregnancies in this study. This percentage has since increased to 49.8% in 2020 (21).

During the same period, a cohort of healthy term-born infants (37–42 weeks birth GA) were recruited on the postnatal wards as part of a randomised clinical trial of a massage intervention (ENRICH study—Clinicaltrials.gov NCT03381027). The non-intervention arm of this clinical trial served as the term control (TC) group for the current study. Parents of infants with no metabolic or genetic anomalies, not requiring specialist ongoing care and not requiring admission to the neonatal unit, were invited to participate. The TC group excluded infants from multiple pregnancies.

### Outcome

As per hospital criteria, cranial ultrasounds (CRUS) were routinely performed on MLP infants <1,500 g or if clinically indicated.

Infants from both groups (MLP and TC) returned to the INFANT Early Life Lab for a neurodevelopmental assessment at 18 months PMA (+2 weeks). Identifying, quantifying and predicting suboptimal development requires a valid and reliable standardised neurodevelopmental assessment tool. The Griffiths lll mental developmental scale (MDS), is a norm-based validated neurodevelopmental assessment and was used in this study. The Griffith's III was validated on a representative sample of infants born in Ireland and the UK (22). Children were seated at a low table beside their parent/guardian during the assessment which took on average approximately 45 min. The same group of experienced assessors performed assessments for both study groups. Due to the nature of the study and the assessment used, we were unable to blind assessors to group membership.

The Griffiths lll provides an overall general neurodevelopmental score and an individual profile of strengths and weaknesses across five areas or subscales of neurodevelopment (23). Subscales of development include: Foundations of Learning (curiosity, communication creativity, learning and memory), Hearing/speech and communication, Eye-hand coordination coordination, Personal–social performance, and Gross Motor development. Each subscale is equally weighted and is a complete and separate scale in itself. Developmental scores were calculated for each subscale of development based on the mean score obtained for each group (24). Input from primary caregivers is required for this assessment with some subscales requiring more input than others (25). Caregiver input confirms the infant's ability to carry out specific activities in the home that may not be completed on the day of assessment. In calculating scores achieved with Griffiths lll, “*ratio transformation”* is used which divides developmental age in months by psychologists age giving rise to slightly different means and standard deviations for each subscale of development (26).

Using the Irish Central Statistics Office criteria (27) we categorised employment as: professional, lower professional, manager/technical/administration, non-manual or skilled. Data relating to maternal occupation for the TC group data was obtained from a maternal questionnaire completed as part of the original clinical trial. Data relating to maternal occupation of the MLP group was obtained from the maternal electronic health record system if documented. No data was available on paternal occupation.

### Research ethics

Ethical approval was granted by the Clinical Research Ethics Committee (CREC) of the Cork Teaching Hospitals. Parents of all infants included gave informed consent for participation and publication of the results. Consent was sought as per good clinical practice (GCP) ICH GCP E6 (R2) guidelines. Data that was recorded followed General Data Protection and Regulations (GDPR 2016/679).

### Statistical analysis

Demographic and clinical characteristics of the MLP and TC groups were described using the median and inter-quartile range (IQR) for continuous variables and frequencies and percentages for categorical variables. Comparisons of demographical variables between the MLP and TC groups were made using the Mann–Whitney *U*-test for continuous variables and using the chi-squared test or Fisher's exact test (in the case of small expected counts) for categorical variables.

Griffiths DQ scores were described using the mean and standard deviation (SD) and the independent samples *t*-test was used to compare scores between MLP and TC groups. Cohen's *d* formula was used to determine the standardized mean difference between the MLP and TC groups. A *d* of 0.2 was considered a small effect size, 0.5 a medium effect size and 0.8 a large effect size. To adjust for the potential confounding effect of sex, linear regression analysis was performed with sex and group included as independent variables in the model. As the type of pregnancy (singleton vs. multiple) was also a potential confounder and there were no multiples included in the TC group, linear regression analysis, unadjusted and adjusted for sex was also performed using the singleton infants only.

Within the MLP infant group analysis, a comparison of DQ scores between two groups (e.g., sex and pregnancy type) was performed using an independent samples *t*-test and more than two groups [e.g., gestational age groups (week)] using a one-way ANOVA. Infants in the MLP group were defined as having a normal outcome if their general development DQ score was within 1 SD of the mean score of the healthy term control group [mean (SD): 119 (10)].

Statistical analysis was performed using IBM SPSS Statistics (version 25.0, IBM Corp, Armonk, NY, USA). All tests were two-sided and a *p*-value <0.05 was considered statistically significant.

## Results

One hundred and one MLP infants and ninty seven term control (TC) infants were recruited at birth and enrolled in this study. The median (IQR) birth GA (weeks) was 34.0 (33.0–35.0) for the MLP group and 39.9(38.9–40.9) for the TC group. There was no difference in maternal age between groups (*p* = 0.785). As expected the TC group had significantly higher birthweight and larger head circumference than the MLP group (*p* < 0.001 for both) (Table 1). A higher percentage of infants in both groups were male (MLP 52.5% and TC 57.7%). Participants from TC and MLP groups were predominantly Irish Caucasian (89.7% and 93.1% respectively) which reflects the local population. Whilst 96.7% of mothers of TC group were employed, maternal occupation was not documented for two participants (2.2%). In the MLP group, 74.7% of mothers were employed with maternal occupation not documented in 18.7% of the group. There was no significant difference in employment category between TC and MLP infant groups (*p* = 0.784).

**Table 1:** Demographics.

Birth	Term (*n* = 97)	MLP (*n* = 101)	*p*-value^a^
	median (IQR)	median (IQR)	
Maternal age(years)	35.0 (32.0 to 37.0)	35.0 (31.0 to 38.0)	0.785
Gestational age at birth (weeks)	39.9 (38.9 to 40.9)	34.0 (33.0 to 35.0)	
Birth weight (kg)	3.60 (3.29 to 3.83)	2.10 (1.88 to 2.40)	<0.001
Head circumference(cm)	35.20 (34.55 to 36.10)	32.00 (30.80 to 32.90)	<0.001
	*n* (%)	*n* (%)	
Sex			0.457
Male	56 (57.7)	53 (52.5)	
Female	41 (42.3)	48 (47.5)	
Nationality			0.435
Irish caucasian	87 (89.7)	94 (93.1)	
Non-Irish caucasian	8 (8.2)	7 (6.9)	
Asian/African	2 (2.1)	0 (0.0)	
Pregnancy			
Singleton	97 (100.0)	53 (52.5)	
Multiple	0 (0.0)	48 (47.5)	
18 months	Term (*n* = 92)	MLP (*n* = 75)	
	median (IQR)	median (IQR)	*p*-value^a^
Weight (kg)	11.50 (10.70 to 12.60)	11.36 (10.75 to 12.40)	0.518
Height (cm)	83.00 (80.00 to 85.00)	81.65 (79.43 to 84.00)	0.079
Head circumference (cm)	48.80 (47.53 to 49.50)	48.00 (47.00 to 48.50)	<0.001
Chronological age ^b^	18.46 (18.17 to 18.72)	19.68 (19.32 to 20.50)	<0.001
Adjusted age on assessment ^b^	18.00 (18.00 to 18.00)	18.0 (18.0 to 20.0)	<0.001
	*n*(%)	*n*(%)	
Sex			0.091
Male	55 (59.8)	35 (46.7)	
Female	37 (40.2)	40 (53.3)	
Nationality			0.010
Irish caucasian	83 (90.2)	75 (100)	
Non-Irish caucasian	7 (7.6)	0 (0.0)	
Asian	2 (2.2)	0 (0.0)	
Pregnancy			
Singleton	92 (100)	38 (50.7)	
Multiple	0 (0.0)	37 (49.3)	
Maternal occupation			0.070^c^
Unemployed	0 (0.0)	2 (2.7)	
University student	1 (1.1)	3 (4.0)	
Not documented	2 (2.2)	14 (18.7)	
Employed	89 (96.7)	56 (74.7)	0.784^d^
Professional	57 (64.0)	40 (71.4)	
Lower professional	2 (2.2)	2 (3.6)	
Manager/technical/admin	14 (15.7)	8 (14.3)	
Non-manual	10 (11.2)	5 (8.9)	
Skilled	5(5.6)	1(1.8)	
Not documented	1(1.1)	0(0.0­)	

MLP, moderate to late preterm infants; IQR, interquartile range; kg, kilogram; cm, centimetre.

A *p-*value <0.05 is considered statistically significant.

Missing data as not recorded in electronic health records or failure to obtain at 18 months.

In MLP group missing data includes: head circumference at birth (*n* = 2) and at 18 months includes: weight (*n* = 4), height (*n* = 1), head circumference (*n* = 6), and maternal occupation (*n* = 14).

In term control group missing data includes: head circumference at birth (*n* = 4) and at 18 months includes weight (*n* = 4), height (*n* = 3), head circumference (*n* = 16), and maternal occupation (*n* = 2).

^a^ From the Mann–Whitney *U*-test for continuous variables and from the Chi-squared test or Fisher's exact test for categorical variables.

^b^ Delay in 18 months assessment for MLP (*n* = 14) infants due to Covid restriction.

^c^ From Fisher's exact test based on comparisons between groups with “not documented” group excluded.

^d^ From Fisher's exact test based on comparisons between employment groups with ‘not documented’ group excluded.

Within the MLP group, 52.5% (53/101) were from singleton pregnancies. MLP infants from a singleton pregnancy(*n* = 53) had significantly higher birthweight than infants from a multiple pregnancy(*n* = 48) (median (IQR): 2.27(1.95–2.65) kg vs. 1.97(1.78–2.14) kg, *p* < 0.001); however, this difference in weight between infants from a singleton(*n* = 36 and multiple pregnancy(*n* = 35 did not persist at 18 months (median(IQR): 11.29(10.80–12.49) kg vs. 11.37(10.60–12.24) kg, *p* = 0.666). In the MLP group, nine infants had a birth weight < 10th centile for GA i.e., small for gestational age. Cranial ultrasounds were performed on three infants (all <1,500 g), as per local guidelines, all of which were normal prior to discharge.

At 18 months (+2 weeks) PMA 74.3% (75**/**101) of the MLP infant group returned for follow up. The large fall out rate (*n* = 26) may in part have been associated with Covid 19 and some families chose to withdraw and some parents were not contactable or known to have moved overseas. Assessments were delayed for a number of MLP infants (*n* = 14). These infants were assessed at 22 months PMA as soon as restrictions were lifted and Griffiths scores were adjusted according to PMA. There was no significant difference in birth demographics between MLP infants who were lost to follow up and those who returned at 18 months. Follow up of the TC group was completed prior to the global pandemic and hence study retention was not impacted by same. In the TC group 94.8% (92/97) returned for a Griffiths developmental assessment at 18 months(+2 weeks) (Table 1).

### Comparison of neurodevelopmental outcome between MLP and TC groups

General development DQ scores and the subscales of development scores for the MLP and TC groups are described and compared in Table 2. The mean(SD) GD DQ score in our healthy TC group was 119.45 (10.78) and the MLP infant group was 112.12(13.67). DQ values for the TC group were significantly higher than published values of 109(19). These higher scores seen in the control group may be due to the fact that these infants were low risk, term infants with normal Apgar scores and did not require admission to the NICU. It may also be due to the fact that this is one of the first studies published using the Griffiths III and may present valuable data on real world use in the Irish population.

**Table 2:** Neurodevelopmental outcome of term control (TC) and moderate to late preterm (MLP) infant group at 18 months.

	Term control (TC) Group (*n* = 92)	Moderate to late preterm (MLP) Group (*n* = 75)			
	mean (SD)	mean (SD)	Difference in means (95% CI)^a^	*p*-value^b^	Cohens *d*^c^
Subscale A DQ Foundations of Learning	112.46 (11.92)	106.45 (14.30)	6.00 (2.00 to 10.01)	0.004	0.460
Subscale B DQ, Language and Communication	107.99 (14.85)	103.97 (17.19)	4.02 (−0.88 to 8.91)	0.107	0.252
Subscale C DQ, Eye - hand coordination	116.16 (10.75)	108.36 (13.45)	7.80 (4.11–11.50)	<0.001	0.648
Subscale D DQ, Personal, Social and Emotional	119.04 (8.17)	113.56 (10.51)	5.48 (2.63–8.34)	<0.001	0.590
Subscale E DQ, Gross Motor	130.30 (13.58)	120.85 (14.94)	9.45 (5.09–13.81)	<0.001	0.665
GD DQ, General Development	119.45 (10.78)	112.12 (13.67)	7.33 (3.59–11.06)	<0.001	0.602

DQ, developmental quotient score; SD, standard deviation; CI, confidence interval.

A *p*-value <0.05 is considered statistically significant.

^a^ Difference is TC-MLP.

^b^ From independent samples *t*-test.

^c^ Cohen's *d* formula was used to determine the standardized mean difference between the MLP and TC groups.

The mean DQ score in all subscales of development and overall general development were significantly higher in the TC group compared to the MLP group with the exception of subscale B: Language and Communication. The greatest differences were in Gross Motor development (*p* < 0.001, Cohen's *d* = 0.665) and Eye-hand co-ordination (*p* < 0.001, Cohen's *d *= 0.648). Being within +/− 1 SD from the mean is considered normal for data.

#### Adjustment for sex

As sex was a potential confounder in the relationship between DQ scores and group, an adjusted analysis was performed (Table 3). Larger differences were found between the two groups in the adjusted analysis compared to the unadjusted analysis.

**Table 3:** Comparison of mean scores at 18 months between TC and MLP infants unadjusted and adjusted for sex.

	Unadjusted analysis	*p*-value^b^	Analysis adjusted for sex	*p*-value^c^
	Difference in means(95% CI)^a^		Difference in means(95% CI)^a^	
Subscale A DQ Foundations of Learning	6.00 (2.00 to 10.01)	0.004	6.55 (2.55 to 10.55)	0.001
Subscale B DQ, Language/Communication	4.02(−0.88 to 8.91)	0.107	5.30 (0.58 to 10.02)	0.028
Subscale C DQ, Eye-hand coordination	7.80 (4.11 to 11.50)	<0.001	8.56 (4.93 to 12.19)	<0.001
Subscale D DQ, Personal/Social and Emotional	5.48 (2.63 to 8.34)	<0.001	6.20 (3.44 to 8.96)	<0.001
Subscale E DQ, Gross Motor	9.45 (5.09 to 13.81)	<0.001	9.76 (5.36 to 14.16)	<0.001
GD DQ, General Development	7.33 (3.59 to 11.06)	<0.001	8.25 (4.63 to 11.87)	<0.001

CI, confidence interval.

A *p*-value < 0.05 is considered statistically significant.

^a^ Difference is TC-MLP.

^b^ From linear regression analysis with group as the independent variable.

^c^ From linear regression analysis with group and sex as the independent variables.

After adjustment for sex, mean scores were significantly higher in the TC group compared to the MLP group for all subscales, including subscale B: Language and Communication (*p* = 0.028).

#### Adjustment for pregnancy type (singleton v multiple) and sex

Pregnancy type was a potential confounder in the relationship between DQ scores and group. As the TC group only included singleton infants, we performed an analysis restricted to singleton infants (*n* = 92 in the TC group and *n* = 38 in the MLP group, Table 4). In the unadjusted and adjusted analysis, mean scores were significantly higher in the TC group compared to the MLP group for the general development DQ score and all subscales of development except for subscale B: Language and Communication.

**Table 4:** Comparison at 18 months between TC (*n* = 92) and MLP (*n* = 38) groups, singleton infants only.

	Unadjusted analysis	*p*-value^b^	Analysis adjusted for sex	*p*-value^b^
	Difference in means (95% CI)^a^		Difference in means (95% CI)^a^	
Subscale A DQ Foundations of Learning	6.09 (1.59 to 10.59)	0.008	6.38 (1.86 to 10.91)	0.006
Subscale B DQ, Language and Communication	0.02 (−5.61 to 5.64)	0.996	0.79 (−4.77 to 6.35)	0.779
Subscale C DQ, Eye-hand coordination	5.95 (1.86 to 10.05)	0.005	6.49 (2.43 to 10.54)	0.002
Subscale D DQ, Personal, Social and Emotional	4.23 (1.19 to 7.27)	0.007	4.72 (1.73 to 7.70)	0.002
Subscale E DQ, Gross Motor	11.57 (6.08 to 17.06)	<0.001	11.80 (6.26 to 17.33)	<0.001
GD DQ, General Development	5.76 (1.69 to 9.83)	0.006	6.39 (2.40 to 10.39)	0.002

CI, confidence interval.

A *p*-value <0.05 is considered statistically significant.

^a^ Difference is TC-MLP.

^b^ From linear regression analysis with group as the independent variable.

^c^ From linear regression analysis with group and sex as the independent variables.

### MLP group only—investigation of variables associated with neurodevelopmental outcome

There were no significant differences in outcomes by birth gestational age group. (Table 5) Female infants outperformed male infants subscale B: Language and Communication (*p* < 0.001), subscale C: Eye-hand coordination (*p* = 0.003), subscale D: Personal, Social and Emotional (*p* < 0.001), and overall general development (GD) (*p* < 0.001). (Table 5) There was no difference in overall general developmental (GD) scores between infants born from singleton and multiple pregnancies (*p* = 0.319). Significant differences by pregnancy type were only evident in subscale B: Language and Communication (*p* = 0.040) in which singleton infants outperformed infants born from a multiple pregnancy. All SGA infants, (four infants from singleton pregnancies and 5 infants from multiple pregnancies) achieved normal Griffiths III General Development DQ score at 18 months.

**Table 5:** Relationships between the degree of prematurity (GA week group), sex, pregnancy type and outcome of MLP infants at 18 months, *n* = 75.

Gestational age group (weeks)	Subscale A: Foundations of Learning	*p*-value^a^	Subscale B: Language and Communication	*p*-value^a^	Subscale C: Eye-hand coordination	*p*-value^a^	Subscale D: Personal, Social and Emotional	*p*-value^a^	Subscale E: Gross motor development	*p*-value^a^	General development	*p*-value^a^
	mean(SD)		mean(SD)		mean(SD)		mean(SD)		mean(SD)		mean(SD)	
		0.978		0.450		0.741		0.573		0.713		0.585
32 (*n* = 12)	108.58 (12.26)		109.17 (7.38)		110.25 (9.72)		116.50 (6.95)		120.50 (13.35)		115.25 (6.09)	
33 (*n* = 13)	106.31 (14.23)		101.54 (20.70)		108.15 (15.69)		114.38 (16.52)		125.08 (14.61)		112.46 (16.86)	
34 (*n* = 28)	105.57 (16.68)		100.14 (18.31)		105.79 (14.20)		111.00 (10.02)		118.29 (18.92)		108.82 (15.14)	
35 (*n* = 10)	105.50 (12.38)		108.70 (20.99)		111.60 (14.86)		115.10 (7.94)		120.20 (8.60)		114.00 (12.98)	
36 (*n* = 12)	107.33 (13.67)		106.42 (13.49)		110.00 (12.02)		114.42 (8.36)		123.17 (10.32)		114.75 (12.69)	
Sex		0.075		<0.001		0.003		<0.001		0.582		<0.001
Male (*n* = 35)	103.31 (16.69)		95.80 (19.38)		103.46 (15.89)		109.26 (12.41)		119.83 (14.49)		106.66 (16.53)	
Female (*n* = 40)	109.20 (11.34)		111.13 (11.00)		112.65 (9.08)		117.33 (6.66)		121.75 (15.44)		116.90 (8.14)	
Pregnancy type		0.959		0.040		0.230		0.298		0.216		0.319
Singleton (*n* = 38)	106.37 (11.46)		107.97 (14.51)		110.21 (10.69)		114.82 (7.48)		118.74 (16.20)		113.68 (10.41)	
Multiple (*n* = 37)	106.54 (16.89)		99.86 (18.89)		106.46 (15.71)		112.27(12.90)		123.03(13.39)		110.51(16.36)	

SD, standard deviation.

A *p*-value <0.05 is considered statistically significant.

^a^ From independent samples *t*-test when a variable has two categories (sex, pregnancy type) and from one-way ANOVA when the variable has more than two categories (GA group).

## Discussion

In two carefully categorised prospective healthy cohorts we have shown that MLP infants had significantly lower scores compared to TC infants in the Griffiths lll MDS at 18 months. This study shows that MLP infants are vulnerable to suboptimal neurodevelopment. There were no significant differences in scores found in Subscale B (Language and Communication) between the two groups (*p* = 0.107) however, when we controlled for sex, a significant difference was evident between the groups (*p* = 0.028). In a study by Putnick et al. inclusive of very preterm infants (*n* = 204), moderate-late preterm (*n* = 276) and term born infants (*n* = 268), language was assessed at 5 months, 20 months, 4 years, 6 years and 8 years of age (28). Very preterm children consistently performed less well than term-born children, with moderate-late preterm children also identified as being at risk for poorer language performance compared to term-born children. By comparison, Perez-Pereira et al. compared language development of low risk preterm infants (*n* = 150, mean GA of 32.62 weeks) and term born children (*n* = 49, mean GA of 39.70 weeks) up to 30 months and concluded that low risk preterm children were not at risk of delayed language development (29).

Both MLP and TC infant groups scored high in Subscale E (Gross Motor). However, statistically significant differences were evident between the groups (*p* < 0.001) with the TC group out-performing the MLP infant group. When the MLP group was subdivided by pregnancy type, differences were evident in Subscale B (Language and Communication) in which MLP infants from singleton pregnancies significantly outperformed MLP infants born from a multiple pregnancy (*p* = 0.040). A systematic review comparing the outcome of infants from twin and singleton pregnancies described differences in language and communication but proposed that the likely cause was due to the parental demands of managing two infants (30). Thorpe identified language delay in twins as mild but prevalent especially in male infants (31). It is not certain if low birth weight in multiple pregnancies may be a potential confounder which is associated with 60% of twin pregnancies (32).

Capobianco et al. compared the outcome of preterm infants (average birth GA of 32 weeks and birth weight of 2,200 g) with no neurological sequela (*n* = 20) and a normal Bayley's lll score(>85) at 18 and 24 months with term born infants (average birth GA of 39 weeks and birth weight of 3,500 g) (*n* = 20). The preterm infant group demonstrated significantly slower language acquisition but these differences were only significant at 16 months (*p* < 0.001) and 18 months (*p* < 0.005) but not at 20, 22, 24 months of age (33). However, in the Copabianco study, assessments were based on chronological age rather than corrected age. Using the Bayley's ll developmental assessment, Gould et al. described differences in scores achieved by preterm infants (*n* = 554) based on chronological age vs. corrected age as 17.3 points lower on the mental scale (79.5 vs. 96.8 respectively) and 11.8 points lower on the motor scale (84.8 vs. 96.6, respectively) at 18 months (34).

In the MLP group, we did not find any significant differences in outcome related to birth GA. This may be because of low numbers at each gestational age group meaning that we were underpowered to examine this effect. In a similar study investigating the neurodevelopmental outcome and social-emotional competence of MLP infants vs. a term control infant group at 2 years, Cheong et al. also found little evidence of an association with gestational age (7).

As infants in both studies had an uncomplicated neonatal stay / hospital stay on post-natal wards, factors such as suboptimal brain development and/or a high frequency of mild brain injuries (35), maternal well-being, obstetric/fetal complications warrant consideration in explaining the differences in developmental outcome between the groups. Pre-eclampsia, male sex and interventional delivery have been identified as antenatal/perinatal risk factors for poor school performance, which Johnson et al. also identified as independent risk factors for cognitive impairment (14).

Other determinants of neurobehavior and intellectual development include level of parental education, socioeconomic status, cultural background and maternal mental health conditions which may be considered additional risks for suboptimal outcome (36–41).

Socioeconomic metrics may be derived from many different aspects including maternal education (40). Maternal education is the most strongly associated dimension of a child's neurocognitive development (42). Detailed data on maternal education were not available for our MLP cohort. Of those employed similar profiles of maternal occupation were seen, with 71.4% of mothers of MLP infants identified as professionals requiring a minimum of a university degree whilst 64.0% of TC group fell into the same category. These high percentages may in part reflect the Organisation for Economic Co-operation and Development (OECD) findings that 60% of women in Ireland between 25 and 34 years and 51% of women between 25 and 64 years have a tertiary education (43). It also may reflect the local community of 210,000 persons from which the cohort was recruited which is home to world leading information technology and software development organisations, large pharmaceutical companies, the largest acute tertiary hospital outside the country's capital and a high ranking university, all of which have high employment of professionals. Finally, it may also be reflective of the professional mothers who agree to take part in birth cohort studies, which is generally higher than the general population. We have reported similar high rates of tertiary education in a previous cohort study (44).

As 18.7% of mothers in MLP group (vs. 2.2% of TC group) had an undocumented occupation this led to employment analysis of a smaller group (*n* = 56) of MLP infants. This may have impacted the differences in percentage of employed professionals in the MLP infant group (71.4%) versus TC group 64.0% (57/89). It is reassuring that the differences in neurodevelopmental outcome are not due to an imbalance in maternal employment between the two groups (*p* = 0.784).

The results of our study are consistent with those of Johnson et al. (*n* = 638 MLP infants and *n* = 765 TC infants) (14) describing female infants outperforming male infants in the MLP group with significantly higher scores in overall general development (*p* < 0.001). However, the Johnson study was based on parental questionnaires in comparison to our study which used a well-validated neurodevelopmental assessment tool.

A gender disparity is evident in the literature with male infants having a greater mortality and respiratory morbidity than female infants which is not explained by neonatal factors (45). In a review of outcomes of male and female preterm infants, O’Driscoll et al. described an increased vulnerability of male preterm infants which they suggest is likely to be multifactorial and perhaps includes hormonal and genetic factors and immunological differences between males and females (46). Romeo et al. described significant gender differences in low-risk preterm infant groups (*n* = 69 very preterm and *n* = 71 late preterm infants) and no gender differences amongst term born infants (*n* = 48) based on the scores achieved in the Bayley's scales of Infant Development, 2nd edition at two years (47). However no gender differences were reported by Romeo et al. at 12 and 24 months using the Griffiths ll standardised assessment tool which the authors suggested was attributed to a higher ratio of female to male participants (F:M = 1.6:1) (48).

The risk of preterm birth and infant mortality rates are higher in multiple pregnancies (49, 50). Almost half of the MLP infants in this study were from a multiple pregnancy (49.3%). General development DQ scores of MLP infants from singleton (*n* = 38) and multiple pregnancies (*n* = 37) at 18 months were comparable (*p* = 0.319). Restricting the analysis to singletons in both groups did not change our findings (Table 4).

Overall cognitive performance of children born moderately preterm (MPT) and MLP have been described as consistently lower in comparison to the term-born group (51). A study by Cheong et al. (7) of MLP and term-born infants concluded that the greatest difference between the groups was in the language domain at 2 years (−11.4 (95% CI, −15.3 to −7.5). However, our study has not shown a statistically significant difference between the MLP and TC groups in the domain of language and communication (*p* = 0.107). These differences may in part be explained by the differences in the timing of the assessment and the assessment tool used; Cheong et al. used the Bayley Scales of Infant Development assessment (Third Edition) at 2 years corrected age.

Fernald and Marchman cite individual differences in language proficiency which can accelerate and abate at different time points for each infant (52). Our single assessment provides a snapshot of language development at one-time point at 18 months, which may be too early to detect significant language delay and the clinical relevance of our findings on long term outcomes has yet to be established. We accept that neurodevelopmental deficits may be quite subtle and frequently not become evident until preschool or school-age years and may change positively or negatively at an older age (53). All impairments, irrespective of degree, have the potential to limit professional options, life opportunities and overall quality of life.

In this study, MLP infants had significantly lower scores in the personal, social and emotional subscale of development (*p* < 0.001) with a moderate effect size (Cohen's *d *= 0.590) than the TC group. Research has shown an increased risk of delayed social competence, with social behavioural issues higher amongst MLP infants in comparison to term-born infants (54). MLP infants also have a predisposition to more challenging behavioural issues with an increased risk of hyperactivity and attention deficit hyperactivity disorder (ADHD) (51). However, the association between prematurity and hyperactivity has not been firmly established (55). Children with learning, social, motor and sensory impairments that challenge their performance prior to or during school-going years may be considered to have special educational needs (SEN). Increased SEN are well described in MLP -born groups (56). Odd et al. identified MLP infants as being 50% more likely to have SEN requiring support in comparison to healthy term-born infants (57).

A strong correlation has been shown between prematurity and a diagnosis of cerebral palsy (CP) with the absolute risk of CP increasing with decreasing birth GA. Pierrat et al. reported that 1.0% of infants born between 32 and 34 weeks GA weeks (*n* = 1,187) had a diagnosis of CP at 2 years of age (58). Although more mature preterm infants generally have better outcomes, MLP infants have a six times higher prevalence of CP than children born at term (7 per 1,000 vs. 1.1 per 1,000 live births) (59). This may be related to the incidence of brain lesions in MLP infants described previously on cranial ultrasound (35) the clinical relevance of which warrants further study including long-term follow-up.

Our study has shown that the greatest difference between the MLP and TC groups was in the area of gross motor development (*p* < 0.001, Cohen's *d* = 0.665) and eye-hand co-ordination (*p* < 0.001, Cohen's *d *= 0.648) with a moderate effect size for each subscale. In this study no MLP infants had a diagnosis of CP at 18 months based on neurological exam nor were there any concerns about motor impairment. However, mild motor impairment may only become apparent beyond 18 months. Williams et al. suggest that motor impairment (either CP or non-CP e.g. Developmental Coordination Disorder) may be subtle and not evident until early childhood years and may be the most common form of impairment in the preterm population (60).

### Strengths and limitations

This study provides robust data to describe the developmental outcome of a large group of healthy MLP infants. Homogeneity is an important factor for identifying norms for specific groups of preterm children and a major strength of this study. All MLP infants had an uncomplicated journey through the neonatal unit with no neurological concerns and were considered healthy at discharge. Both the MLP and TC groups were recruited over a similar period of time (2017–2018) and infants were assessed by the same group of highly experienced assessors at the same time point of 18 months PMA with the exception of some MLP infants with a delayed assessment due to Covid restrictions.

Assessing infants based on PMA or corrected age decreases the transient developmental gap that MLP infants experience whilst they catch up to infants born at term (61). A failure to correct for GA at birth has implications for intervention programmes. Differences in scores may assist parents/schools in decision-making relating to the age of starting school. Placing the child in the correct year for PMA rather than the school year based on calendar age has led to greater academic success (57). The MLP infant group included infants from singleton and multiple pregnancies. With close to half MLP infants born from a multiple pregnancy, inclusion of multiples in our MLP group ensures a true representative group.

A neurological exam was normal in the MLP group and infants were considered healthy prior to discharge. At the time of this study (2017–2018) healthy preterm infants > 32 weeks had a cranial ultrasound performed if < 1,500 g (*n* = 3) or neurological concerns (*n* = 0) as CRUS was not standard practice in the care of MLP in our unit at the time of study recruitment. Boswinkel et al. identified minor brain lesions on CRUS and /or MRI in over 30% of MLP infants (35). As this was not standard practice in our unit at the time of study recruitment, we cannot rule out the presence of minor brain abnormalities in the MLP group, which may have influenced neurodevelopmental outcome. Neurological status was based on clinical neurological examination. Infants did not have a structured neurological exam such as the Hammersmith Infant Neurological Exam at follow up appointments in neonatal clinics. This is a limitation of this study. However, infants did have a clinical neurological examination by a neonatal consultant in outpatients and if any concerns arose, infants would have been referred for further assessment and/or intervention.

Participants were recruited from a single research site reflecting the local population. As a result, there was a lack of diversity in our population which limits the generalisability of our findings.

Follow up of the TC group was completed prior to Covid restrictions. These restrictions may be considered an unavoidable limitation of this study resulting in a delay in the assessment of a small group of MLP infants (*n* = 14). Multiple births were an exclusion-criteria for the TC group. All MLP participants were admitted to the neonatal unit to the exclusion of those cared for in postnatal wards. This may have had implications in terms of the level of stress or sensory exposure for the MLP infant in comparison to the TC group (62).

Research has shown that neurodevelopmental delay is more prevalent amongst MLP infants from disadvantaged socioeconomic groups. As this data was not available in our study, we utilised maternal education levels (where documented) as a proxy for socioeconomic status. This data was not recorded for 18.7% of MLP infants. Another possible limitation includes the potential for bias in scoring infants in neurodevelopmental assessments as assessors were not blinded to the study group.

### Future research

There is limited data available on long-term neurodevelopmental outcome of MLP infants with an uncomplicated neonatal stay and a normal neurological exam on discharge. Institutional practices relating to pre-discharge screening including CRUS or MRI and post discharge neurological surveillance vary (16). Standardising screening practice based on findings from multicentre longitudinal research studies with expert input would identify best practice for follow up of this at risk group.

Accuracy in determining the presence and degree of suboptimal neurodevelopment is dependent on the reliability and validity of the assessment tool, the assessment time point and the experience of the assessors. Hence identifying a screening tool(s) and screening intervals appropriate to all infant groups is a priority. Surveillance of preterm infants presents a unique opportunity for early diagnosis of neurodevelopmental delay and intervention with targeted therapies. As neurodevelopmental delay can be transitory or sustained we do not know what these suboptimal scores at 18 months will mean for this MLP infant group in the future. Early assessment is not always predictive of school-age cognitive functioning, long-term learning disabilities or academic achievement (63). Ongoing neurodevelopmental surveillance and sequential screening are especially pertinent to infants who have a previous abnormal assessment.

## Conclusion

MLP infants are at higher risk of suboptimal outcome at 18 months in comparison to term-born infants. This study highlights the effect of moderate to late preterm birth on neurodevelopmental outcome. Our findings support the need for a broader effort for longitudinal surveillance of the MLP infant group including those who appear healthy.

## Data Availability

For this current study, it is not possible to share data sets. The clinical data are collected under written proxy consent from the participants' guardians/parents that did not include permission for sharing or open data. To be allowed to share these data under Irish Health Research Regulations we are required to re-consent families or obtain approval from the Health Regulation Consent Declaration Committee.
